# Diagnostic TCD for intracranial stenosis in acute stroke patients: experience from a tertiary care stroke center in Karachi, Pakistan

**DOI:** 10.1186/s13104-015-1289-3

**Published:** 2015-08-11

**Authors:** Ayeesha Kamran Kamal, Hasan Rehman, Nasir Mustafa, Bilal Ahmed, Mohammad Jan, Faisal Wadivalla, Syed Kamran

**Affiliations:** Stroke Service, Stroke Fellowship Program, International Cerebrovascular Translational Clinical Research Training Program, Fogarty International Center and The National Institute of Neurologic Disorders and Stroke, Section of Neurology, Department of Medicine, Aga Khan University, Stadium Road, Karachi, 74800 Pakistan; Stroke Service, Section of Neurology, Department of Medicine, Aga Khan University, Karachi, Pakistan; Epidemiology and Biostatistics, Section of Neurology, Department of Medicine, Aga Khan University, Karachi, Pakistan; Data Management Stroke Service, Section of Neurology, Department of Medicine, Aga Khan University, Karachi, Pakistan

**Keywords:** Transcranial Doppler, Intracranial stenosis, Magnetic resonance angiography, Cerebral infarction prevention, Cerebrovascular accident

## Abstract

**Background:**

Stroke is a common cause of morbidity and mortality around the world. Intracranial large artery atherosclerosis (ICAD) is a frequent etiology of stroke in the South Asian population. There is a need for widely available screening tools to identify patients that are at high risk of stroke due to ICAD for aggressive risk management. This study describes the experience of using the transcranial Doppler (TCD) as a screening tool for this purpose at a tertiary care hospital in a developing country.

**Methods:**

86 Patients admitted with stroke due to ICAD underwent TCD for six arteries (Right and left middle cerebral arteries, right and left anterior cerebral arteries, right and left posterior cerebral arteries) in addition to the magnetic resonance angiography (MRA) that is done routinely at the stroke center. Arteries were labeled with either <50 or >50% stenosis by TCD using two separate criteria. These findings were compared with those from the MRA which was used as the gold standard. The proportion of patients that had complete exams (all six arteries insonated by TCD) was reported. The success rate of each TCD criteria in detecting arteries with >50% stenosis was also calculated.

**Results:**

There was an attempt to visualize 516 arteries (86 patients with 6 arteries each) of which 375 (72.7%) were successfully insonated. 38 of the 86 (55.8%) patients had complete examinations. MRA reported 43 (8.3%) arteries as stenosed >50%. The TCD did not categorize any artery as stenosed using either criterion and hence failed to classify any stenosed artery correctly. The positive predictive and sensitivity was 0 for this study and the negative predictive value was 93.3%.

**Conclusions:**

This study indicates the poor sensitivity of TCD to be a reliable screening tool for the presence of ICAD in the South Asian population in a real life clinical setting.

**Electronic supplementary material:**

The online version of this article (doi:10.1186/s13104-015-1289-3) contains supplementary material, which is available to authorized users.

## Background

Stroke is a leading cause of morbidity and mortality worldwide resulting in an estimated 4.4 million deaths each year [1]. Large vessel intracranial atherosclerotic disease (ICAD) is believed to have a higher frequency among the South Asians [2–7] with one study [8] reporting it to be responsible for at least 30–50% strokes among the Asian population.

The pathophysiology of ischemic stroke revolves around atherosclerotic plaque formation over a number of years in the arteries of the Circle of Willis before the final cerebrovascular event. This event is presumably caused by a rupture of that plaque or progressive hemodynamic stenosis leading to decompensated low flow state with failure of the collaterals [9, 10].

Regardless of the mechanism of stroke, prevention is the key to reduce mortality [11]. Studies such as WASID study [12] have identified medications that are beneficial in high risk patients. However, to identify intracranial stenosis there need to be screening tools that are not only effective but also widely available at an affordable cost to make them relevant to low income countries where this condition is prevalent.

The gold standard to detect large artery intracranial stenosis is conventional cerebral angiography [13] but the cost, unavailability and risk of stroke [14–17] make it impractical for screening purposes. Similarly magnetic resonance angiography (MRA) is not considered a viable option due to its high cost and availability issues [18]. Due to its wide availability, significantly less cost and non-invasiveness, the transcranial Doppler sonography (TCD) is considered a potentially valuable screening tool [19]. Previous studies that have looked at this diagnostic modality have shown encouraging results by reporting negative predictive values and sensitivities comparable to that of the MRA [20].

However, the majority of these studies have been performed on the Western population. Hence, this study aims to explore the viability of the TCD in this region as a screening tool to detect intracranial atherosclerotic disease in a real life low middle income country clinical setting.

## Methods

### Study setting

This study was conducted in a tertiary care hospital in Karachi, a metropolitan city located in the south of Pakistan with a multiethnic population of more than 13 million persons [21]. The hospital has its own stroke team and is equipped with MRA and TCD facilities along with on call radiologists round the clock.

### Selection of participants

All patients admitted to this center after being diagnosed with stroke between January 2012 and January 2013 were eligible for this study. Participants were men and women aged >18 years who presented to the Emergency Department and were subsequently diagnosed with stroke due to atherosclerotic disease via CT scan or MRI. Participants had to consent to TCD in addition to routine MRA which is done as part of standard of care of every patient admitted with stroke at this center.

Patients were excluded from participation if they had concomitant acute coronary syndrome or any extracranial vascular disease. Any patient who refused consent was also excluded.

### Data collection instruments

Relevant demographic data including, age and gender was extracted from the medical records available.

The subtype of stroke was determined using the causative classification system for ischemic stroke (CCS) [22].

The MRA was interpreted on digital images and the degree of stenosis was calculated using the following equation [13]:

$$ \% {\text{ stenosis}} = \left[ { 1- \left( {{\text{D}}_{\text{stenosis}} /{\text{D}}_{\text{normal}} } \right)} \right] \times 100 $$

Using the degree of stenosis, the artery was then classified as normal or with mild atherosclerotic changes, moderately stenosed and severely stenosed or occluded. A normal artery had <30% stenosis, a moderately stenosed artery had 30–50% stenosis and a severely stenosed artery was defined as an artery with >50% stenosis. The severely stenosed artery was used as the outcome of interest since the WASID study [12] had defined this as the critical value beyond which there is proven benefit of antiplatelet therapy. The MRA was used as the gold standard since catheter angiography was considered too invasive for the purpose of this study.

All the TCDs were performed and reported by the same sonographer who had received training in this field. He was blinded to the MRA findings and was required to report the peak velocity (PV) and the time averaged mean of the maximum velocity (MV) for the following arteries: the right middle cerebral artery (RMCA), the left middle cerebral artery (LMCA), the right anterior cerebral artery (RACA), the left anterior cerebral artery (LACA), the right posterior cerebral artery (RPCA) and the left posterior cerebral artery (LPCA). The vertebral and the basilar arteries were excluded.

Using the TCD, intracranial stenosis was categorized as >50 or <50%. There were two criteria that were used for this purpose. The first criterion used the mean velocity (MV) while the second criterion used the peak velocity (PV). Both criteria were used independently and results using each criterion are reported.

Using the MV, any artery with a time averaged mean of the maximum velocity of >100 m/s was considered to have >50% stenosis. This value was chosen taking into consideration the findings of the SONIA trial [20].

Using the peak velocity to determine the degree of stenosis, the criterion verified by Baumgartner et al. [23] was used which was slightly more complex. The cut offs for the peak velocity for >50% stenosis were as follows: for the MCAs >220 cm/s; for the PCAs >145 cm/s; For the ACAs >155 cm/s.

As in the SONIA trial [20], the standards set by the Intersocietal Commission for the Accreditation of Vascular Laboratories (ICAVL) [24] were followed for the TCD. Details of these standards are given in Additional file 1: Appendix 1.

### Statistical considerations

The findings of the MRA were compared to those from the TCD to calculate the sensitivity, the positive predictive value and the negative predictive value of the TCD.

Sensitivity = true positive/(true positive + false negative) [25].

Negative predictive value = true negative/(true negative + false negative) [25].

All data was entered and analyzed via SPSS v19. Statistical analysis were reviewed by a biostatistician.

### Ethical considerations

Prior to obtaining data, the study protocol was approved by the Ethical Review Committee of the Aga Khan University Hospital. No financial incentives were provided to any study participant. Written informed consent and verbal assent was given by all participants or their legal surrogate respondents prior to the investigation.

## Results

There were a total of 86 patients who were enrolled in the study. 45 (52%) were male while 41 (48%) were female. The mean age for male patients was 57 (SD: 16) while the mean age for female patients was 60 (SD: 14). 97.7% patients (n = 84) had suffered from an ischemic stroke while only 2.3% (n = 2) had a hemorrhagic stroke.

Among the 86 patients enrolled in the study, all 6 arteries (LACA, RACA, LMCA, RMCA, LPCA, RPCA) were found normal in 55 (63.9%) of the patients as detected by the MRA while the remaining 31 patients had at least one diseased artery.

In all, 516 arteries (86 patients with 6 arteries each) were looked at and the MRA reported 407 (78.9%) of the arteries to be either normal or with mild atherosclerotic irregularity, 66 (12.8%) had moderate stenosis and 43 (8.3%) were severely stenosed or completely occluded. The highest proportion of severely stenosed arteries was in the RMCA (17.4%, n = 15) followed by the LMCA (9.3%). Detailed findings of the MRA are given in Table 1.

**Table 1:** Magnetic resonance angiography findings

	Normal/mild atherosclerotic changes (<30% stenosis)	Moderately stenosed (30–50% stenosis)	Severely stenosed (>50% stenosis)
Right middle cerebral artery	60 (69.8%)	11 (12.8%)	15 (17.4%)
Left middle cerebral artery	67 (77.9%)	11 (12.8%)	8 (9.3%)
Right anterior cerebral artery	71 (82.6%)	10 (11.6%)	5 (5.8%)
Left anterior cerebral artery	70 (81.4%)	11 (12.8%)	5 (5.8%)
Right posterior cerebral artery	70 (81.4%)	12 (14.0%)	4 (4.7%)
Left posterior cerebral artery	69 (80.2%)	11 (12.8%)	6 (7.0%)
Overall	407 (78.9%)	66 (12.8%)	43 (8.3%)

Of the 86 patients, 38 (44.2%) had complete examinations where all 6 arteries of interest (LACA, RACA, LMCA, RMCA, LPCA, RPCA) were seen, 45 had incomplete assessments where at least one but not all arteries were visualized and there were 3 patients, all women, in whom not a single artery was visualized. Overall, there was an attempt to visualize a total of 516 arteries in 86 patients (6 arteries in each patient) through the TCD but they were successful in insonating only 375 (72.7%) arteries. The insonation failure in men was 19.6% (n = 53) while it was 35.7% (n = 88) in women. The arteries most successfully insonated were the MCAs (91.7% RMCAs and 89.5% LMCAs) followed by the ACAs (65.1% LACAs and 68.6% RACAs) while the PCAs were insonated least successfully (61.6% in right 60.5% in left). Insonation success rate for each artery is given in Table 2.

**Table 2:** The proportion of successful and unsuccessful insonations in each artery

	Successful	Unsuccessful
Right middle cerebral artery	78 (91.7%)	8 (9.3%)
Left middle cerebral artery	77 (89.5%)	9 (10.5%)
Right anterior cerebral artery	59 (68.6%)	27 (31.4%)
Left anterior cerebral artery	56 (65.1%)	30 (34.9%)
Right posterior cerebral artery	53 (61.6%)	33 (38.4%)
Left posterior cerebral artery	52 (60.5%)	34 (39.5%)
Total	375 (73.7%)	141 (27.3)

The findings of the sonography were categorized both via the peak velocity as well as the mean velocity to categorize the degree of stenosis as assessed by the TCD. Regardless of the criteria used, no artery was labeled as having >50% stenosis by the TCD. This essentially gives us sensitivity and positive predictive values of 0% for all arteries using either criterion. Similarly both criteria also had an identical negative predictive value (93.3%). A brief comparison on MRA and TCD findings is given in Table 3.

**Table 3:** Comparison of transcranial Doppler and magnetic resonance angiography findings

TCD	MRA	
	>50% stenosis	<50% stenosis
>50% stenosed	0	0
<50% stenosed	25	350
Failed to insonate	18	123
Total	43	473

The proportion of stenosed vessels among arteries that were not successfully insonated was 12.7% which is higher than the proportion of stenosed or occluded vessels (8.3%) among the visualized arteries.

## Discussion

With the abundant data available on the prevalence and the magnitude of ICAD [6, 26–29] it is important to look at screening and diagnostic modalities that can be effective in reducing morbidity and mortality. This is one of the earlier studies to explore the use of intracranial Doppler among the South Asian population. Previous studies [20, 30–33] in other parts of the world regarding the TCD have had promising results. Although the sensitivity has been reported to be as low as 65% in some cases [20], a negative predictive value of over 90% would suggest that the Doppler can be used to screen out patients who would benefit from aggressive risk management [34]. Unfortunately, this study suggests the Doppler ultrasound is an ineffective tool for this purpose in the South Asian setting when done in a pragmatic fashion in real life patients admitted for stroke.

Since only 38 of the 86 patients had complete examinations, TCD appears to be an unreliable screening tool since most patients will then have to undergo further evaluation based on inconclusive results. Although previous studies [35, 36] have reported insonation failures, these failures are estimated to be between 10 and 30%. Hashimoto et al. [37] reported a failure to visualize temporal windows in 29% of the subjects when he studied the Japanese population while another study, also in Japan, by Itoh [38] had a failure rate of around 22.8%. Similarly, when Seidel used the color coded doppler in the German population, he failed to visualize the intracranial arteries in 20% of cases [39]. One study in 2011 [40] in the United States reported an insonation failure of around 22.2% in 99 patients Interestingly, the study mentioned above quoted higher insonation failure rates for African Americans (47%) compared to whites (15%) when it used multivariate analysis. This would support the belief that difficulties that have been documented [19] using TCD, such as difficulty in visualizing temporal windows and fixating probe positions, are more applicable to certain populations compared to others and more specifically Asians and African Americans as compared to Caucasians.

What is also of note however, is that most studies [20, 33, 41–44] that have studied the TCD have primarily looked at the MCAs while only some have studied the vertebrobasilar arteries [32], the PCAs [32, 45, 46] or all the arteries that were included in this study [35, 47]. When we look at the failure rate of MCAs in our study (<10%), it does become comparable to the other studies mentioned earlier. And so perhaps the only artery that had any results was the MCA but even this does not seem feasible.

The rate of insonation failures was higher in women as compared to men. Although this finding has not been encountered commonly, there has been at least one previous study [40] that reported a higher insonation failure rate in women compared to men. On the other hand, Hashimoto et al. [37] reported a much higher (84 vs. 52%) rate of successful insonations in men compared to women.

The most significant result pertaining to the outcome of the ultrasound was the inability of the TCD to correctly detect any vessel as stenosed or occluded. This would rule out the TCD as a screening tool since it fails to identify high risk patients that need to be managed. The two criteria that were used to classify stenosis TCD findings, the PV and MV, both reported negative predictive values of 93.3%. This would seem to be in line with previous studies [20] but it is in fact a reflection of the proportion of normal vessels and their of correct classification Hence, this does not have any importance in relation to identifying diseased vessels. This study therefore cannot be used to establish the superiority of one criterion over another nor does this have any importance when assessing the TCD as a screening tool.

This study is one of the first to look at the TCD in the South Asian population. However, it is a limited observation in a single center with a trained operator who had standardized training and audit. The strokes were classified with rigor and the images reviewed with MRA and radiology. Under these settings and clinical conditions it appears that the TCD is not a useful tool at all. Whether broad based population studies on community based patients would be different is an area of further investigation and these finding are limited to an inpatient type setting.

## Conclusions

With the rapidly growing morbidity and mortality rates of stroke in this region, screening tools need to be devised that can identify high risk patients so that strokes may be prevented. This study evaluated the TCD as a potential screening tool for this purpose in a hospital setting.

## Additional file

Additional file 1:
**Appendix 1.** Reference standards for TCD performance.

## Abbreviations

ICAD: large artery intracranial atherosclerosis
TCD: transcranial Doppler
MRA: magnetic resonance angiography
WASID: warfarin aspirin symptomatic intracranial disease
SONIA: the stroke outcomes and neuroimaging of intracranial atherosclerosis
CCS: causative classification system for ischemic stroke
PV: peak velocity
MV: time averaged mean of the maximum velocity
PCA: posterior cerebral artery
MCA: middle cerebral artery
ACA: anterior cerebral artery
LPCA: left posterior cerebral artery
LMCA: left middle cerebral artery
LACA: left anterior cerebral artery
RPCA: right posterior cerebral artery
RMCA: right middle cerebral artery
RACA: right anterior cerebral artery
ICAVL: Intersocietal Commission for the Accreditation of Vascular Laboratories
