# Supplementary material for: Diagnostic TCD for intracranial stenosis in acute stroke patients: experience from a tertiary care stroke center in Karachi, Pakistan
Source: BMC Res Notes. 2015 Aug 11;8:341. doi: 10.1186/s13104-015-1289-3 (PMC4531487; doi:10.1186/s13104-015-1289-3)
Supplement: Additional file 1: — Appendix 1. Reference standards for TCD performance. [file 13104_2015_1289_MOESM1_ESM.docx]

**Appendix 1**

**Standardization of TCD**

The standards for technique and equipment set by the Intersocietal Commission for the Accreditation of Vascular Laboratories (ICAVL)[[1](#_ENREF_1)] are to be met. These include:

1. The equipment used must be provided with appropriate Doppler and imaging frequencies adjustable depth and position of the range gate for areas of interest and an adjustable Doppler angle.
2. Provide spectral analysis.
3. Provide audible output, a visual display and a permanent image of the waveform.
4. Examining in 5-mm increments to identify focal mean velocity elevation.
5. Examining in 2-mm increments to identify highest mean velocity.
6. Hard-copy image of highest mean velocity submitted for each vessel insonated.
7. A complete exam shows 6 arteries. (Right and left middle cerebral artery, right and left anterior cerebral artery, right and left posterior cerebral artery)

1. (IAC), I.A.C., *IAC Standards and Guidelines for Vascular Testing Accreditation*, in *Intracranial Cerebrovascular Testing*. 2013. p. 29-32.
